# Health care costs for the treatment of breast cancer recurrent events: estimates from a UK-based patient-level analysis

**DOI:** 10.1038/sj.bjc.6603887

**Published:** 2007-07-24

**Authors:** J Karnon, G R Kerr, W Jack, N L Papo, D A Cameron

**Affiliations:** 1 School of Health and Related Research, University of Sheffield, Sheffield, UK; 2 Western General Hospital, Edinburgh, UK; 3 Novartis Pharmaceuticals UK Ltd, Camberley, UK

**Keywords:** breast cancer, recurrence, costs

## Abstract

Cost pressures and the need to demonstrate cost-effectiveness of new interventions require consideration of the costs of treating disease. This study presents analyses of resource use data covering 199 postmenopausal women who experienced a breast cancer recurrent event between 1991 and 2004 and were treated at the Western General Hospital, Edinburgh. Aggregate (5-year) treatment costs for alternative recurrent events were estimated, as well as the annual costs incurred by patients experiencing contralateral, locoregional, or distant recurrence, who remained alive without further recurrence for a year. The 95% confidence intervals for the 5-year costs of recurrence ranged from £10 000 to £37 000 for locoregional recurrence, and £14 500–£20 000 for distant recurrence. No evidence of significant variations in these costs across time periods between 1991 and 2004 was identified. Annual costs for patients remaining in the same health state showed high initial costs for contralateral and locoregional recurrence, with low costs in subsequent years, while costs associated with distant recurrence declined at a slower rate and plateaued at 4–5 years post-diagnosis. The cost estimates presented in this paper not only inform the magnitude of the resource consequences of breast cancer recurrences, but they are also better suited to informing cost-effectiveness analyses, which have a far greater role in allocating health-care resources.

Breast cancer is the most commonly diagnosed cancer in women – nearly one in three (30%) of all cancers in women occur in the breast, with around 41 000 women diagnosed in the United Kingdom in 2001 and 13 000 women dying from breast cancer in the United Kingdom in 2002 (Breast Cancer Care, 2005). There has been a continuing decline in breast cancer mortality over the last 10 years, which is partly due to the continued development of new therapy options for breast cancer, including the third-generation aromatase inhibitors (letrozole, anastrozole, and exemestane) as the hormonal therapies of choice for postmenopausal patients (Coombes *et al*, 2004; ATAC Trialists' Group, 2005; Goss *et al*, 2005; The Breast International Group (BIG) 1-98 Collaborative Group, 2005). In addition, herceptin and the taxanes have also been shown to improve outcomes in early breast cancer, and all of these treatments will lead to further improvements in survival (Nowak *et al*, 2001; Smith *et al*, 2007).

The acceptance of new therapies by regulatory and/or reimbursement authorities increasingly incorporates evidence regarding their cost-effectiveness and whether the additional health benefits are provided at reasonable additional cost. In the context of a global health budget, health technology appraisal (HTA) bodies such as the National Institute for Clinical Excellence (NICE) in the United Kingdom must compare cost-effectiveness across disease areas. This process requires consideration of long-term costs and effects measured in common units, for example, quality-adjusted life years. The use of mathematical models as the framework for combining information from clinical trials and other sources to project the findings of clinical trials to describe the lifetime costs and consequences of treatment is now commonly accepted (National Institute for Clinical Excellence (NICE), 2004). Model-based economic evaluations require data describing the resources used by patients in a particular health state for a defined period of time; assessment of adjuvant therapies requires the estimation of the treatment costs incurred by patients in the years following the experience of progressive breast cancer events.

The estimation of appropriate cost estimates can be difficult due to the lack of costing studies that report contemporary cost data in a suitable format to inform cost-effectiveness models. Such difficulties are particularly apparent in economic evaluations of breast cancer treatments that have been published from a UK perspective, which have had to rely on estimates of resource use derived from clinicians in the form of expert surveys (Nuijten *et al*, 1999; Brown *et al*, 2001; Karnon *et al*, 2003), or by using resource use estimates from other countries (Karnon and Brown, 2002). Papers that have reported UK-specific breast cancer resource use data present incomplete data, for example, only presenting costs of surveillance (Robertson *et al*, 1995) or data at too aggregated a level that cannot be easily converted to usefully inform economic evaluations (Richards *et al*, 1993; Wolstenholme *et al*, 1998).

This study aims to inform clinicians of the aggregate costs of managing breast cancer recurrence, and to inform health economists of time- and state-specific costs of recurrence to inform the population of cost-effectiveness models. Thus, the objectives of this paper are to estimate:

- aggregate costs over a 5-year follow-up period for patients experiencing contralateral primary tumours, locoregional recurrence, and metastatic recurrence,
- annual costs following a contralateral primary tumour or locoregional recurrence in the absence of metastatic disease, and annual costs following the development of metastatic disease, and
- palliative care costs associated with the terminal phase of metastases.

## MATERIALS AND METHODS

### The data

A database of patient-level resource use was collected at the Western General Hospital, Edinburgh, describing resource use by 199 women with early breast cancer who experienced a recurrence, defined as either a contralateral primary tumour or a relapse of the original breast cancer.

Eight hundred and sixteen women were identified in either the Oncology Department database at Western General Hospital or the Edinburgh Breast Unit (Lothian Surgical Audit) database, aged 50 years or more, who had been diagnosed with T1-3, N0-1, M0 early breast cancer between 1991 and 1998, and whose original treatment included surgery+tamoxifen±radiotherapy (RT)±chemotherapy. The patients included in this study were 199 of those patients identified as having had a recurrence between 1991 and 2004. The records contained in the above two databases were checked for completeness of data, and data not routinely held on these databases, such as number of positive nodes, pathology, size, and oestrogen receptor status, were obtained from databases created for other projects or from a manual study of the case records.

Data on resource use post-relapse were obtained from the Oncology database (treatment), case notes (investigations and so on) and the hospital patient administration system (clinic visits and inpatient nights). In the final database, the dates of all relapses experienced by individual patients were included and up to three types of relapses (contralateral primary tumour, locoregional recurrence, and distant recurrence) were recorded. Resource use relating to surgical episodes, radiotherapy, chemotherapy, and hormonal therapy were presented as the date at which they occurred (surgery and radiotherapy) or the dates at which each episode of hormonal therapy or chemotherapy commenced and completed. Patients could receive multiple regimens of chemotherapy and hormonal therapy, and the periods over which each regimen was administered were recorded. Nontreatment-specific resource use items, such as biopsies, scans, outpatient visits, and others, were recorded as the number of units of each item received by patients in each full year subsequent to the date of their first breast cancer relapse (unless a patient died), for a maximum of 5 years post-relapse. The data did not describe resource use according to time period since last event, only time since the first event.

### Data analysis

Two main sets of analyses were undertaken. First, from a budget impact perspective, the aggregate costs were estimated for the 5 years following an initial diagnosis with a recurrent breast cancer event. This analysis differentiated between the alternative sites of first recurrence, namely contralateral primary tumours, locoregional relapses, and metastases. The second main analysis was undertaken from the perspective of informing model-based cost-effectiveness analyses that describe disease pathways as progression through a series of health states. These analyses estimated the annual costs of recurrence in cases where patients did not experience further events, for example, the costs incurred in the year following a locoregional relapse by patients who do not die or develop metastases within that year. Additional analyses were undertaken that described the health service costs incurred within the final few months of life with metastases (the terminal phase), as well as cost analyses in neighbouring time periods to identify any trends in the observed costs of treating breast cancer. The following sections describe the methods for each of these analyses.

#### Analysis of aggregate 5-year costs

Full resource use data were available to the time of death or to 5-year post-relapse for 161 women. The clinical data were analysed to define Kaplan–Meier curves for survival from contralateral primary tumours, locoregional recurrence, and metastatic recurrence, as well as time to metastatic recurrence from locoregional recurrence to illustrate the clinical characteristics of the patient cohorts. The data were analysed as observed with one exception; women recorded as progressing from locoregional recurrence to metastases within 3 months of their locoregional diagnosis were assumed to have had metastases present at the time of the locoregional diagnosis and were analysed as such.

Resource counts were undertaken for each category of resource use (nontreatment-specific, hormonal therapy, chemotherapy, radiotherapy, and surgery) for all 161 patients to time of death or 5 years after their initial recurrent event. Unit costs were attached to the resources used by each patient in each relapse group, and discounted at 3.5% annually. The mean costs in each group were estimated, as well as bootstrapping the cost estimates for each relapse type to estimate 95% confidence intervals.

#### Analysis of annual costs for patients remaining alive without further progression

The objective of these analyses was to estimate annual resource costs for patients surviving each full year post-relapse for contralateral primary tumour (excluding patients in the year that they die, or experience a locoregional or metastatic recurrence), locoregional recurrence (excluding patients in the year that they die or experience a metastatic recurrence), and distant recurrence (excluding patients in the year that they die). The same assumption regarding women diagnosed with a locoregional recurrence and progressing within 3 months to metastases was made in the annual costs analysis as for the aggregate 5-year costs (see above).

The resource use data were counted by the year in which they were incurred for each category of resource use for all 199 patients included in the dataset. Unit costs were attached to the resources used by each patient in each relapse group, with no discounting. The annual cost data were then sorted into 1 of 15 health states (years 1–5 following either a contralateral primary tumour, locoregional recurrence, or metastases) or defined as incomplete. Incomplete annual cost observations included years in which a patient either died or experienced a new breast cancer event, and hence did not remain in the same health state for a full year.

The mean costs in each of the 15 health states were estimated, as well as bootstrapping the cost estimates for each state to estimate 95% confidence intervals.

#### Terminal care costs

An additional analysis of the data was undertaken to estimate the terminal costs of metastatic disease, the period over which costs of treating breast cancer have previously been noted to rise (Will *et al*, 2000). The terminal phase was assumed to have a duration of 3 months in the base-case analysis, though sensitivity analyses were performed with alternative durations.

For the 130 patients who died following development of distant metastases, resource use was counted over the defined terminal period for the following resource categories: hormonal therapy, chemotherapy, radiotherapy, and surgery. For nontreatment-specific resource use, for which data were only presented by year, resource use in the last recorded year was proportionally divided between the months alive to estimate the mean cost over the terminal phase. To illustrate with a hypothetical woman who lives for 1 year and 9 months with metastases, the terminal-phase (nontreatment-specific) costs were estimated as 3/9 of those costs incurred in the final 9 months of her life. For another women living 2 years and 1 month, the terminal-phase costs were estimated as the costs incurred in the final month, plus 2/12 multiplied by the costs incurred in the previous year.

#### Cost analysis by time period

The final set of analyses involved a comparison of the costs incurred during the first year post-event, by event type, for women experiencing their initial recurrent event within three overlapping time periods: 1991–1995; 1995–1999; and 1999–2004. Resource use was counted to the sooner of the end of the first year or time of death for women relapsing in each time period. Unit costs were attached to the resources used. The mean costs in each group were estimated, as well as bootstrapping the cost estimates for each relapse type and time period to estimate standard deviations. Analysis of variance was then undertaken to identify any significant differences between the time periods.

### Unit cost data

Estimated unit costs for each of the resource use items included in the Edinburgh dataset are described in Tables 1 and 2. The majority of unit costs were derived from the NHS reference costs, which are based on returns from all Hospital Trusts across England and Wales.

## RESULTS

**Figure 1:**
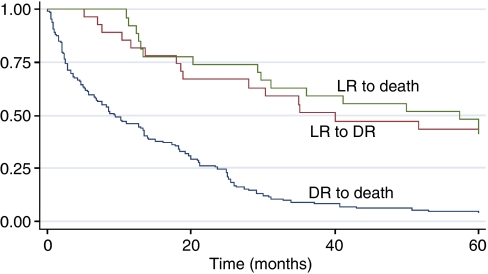
Kaplan–Meier curves for survival and time to metastases.

Table 3 describes the relevant patient characteristics for all patients and for those who relapsed. The median age of the 199 patients who relapsed was 59 years, with a range of 40–82 years. The majority were oestrogen receptor-positive and around half were node negative at primary diagnosis. Figure 1 describes the survival curves for time to metastatic recurrence from locoregional recurrence, overall survival (OS) from locoregional recurrence, and overall survival from metastatic recurrence. The survival curve for patients with only locoregional recurrence reveals a 40% survival rate at 5 years. Following an initial divergence, the locoregional survival curve runs approximately parallel to the time to metastatic recurrence curve, reflecting the impact of metastatic recurrence on survival in patients with locoregional recurrence. The recognised poor survival rate of patients with metastatic recurrence is also reflected in Figure 1.

Table 4 presents some summary data for selected resources for patients with metastatic relapse. This shows that almost 60% of patients diagnosed with metastases received at least one hormonal therapy, and around half received at least one chemotherapy (almost a quarter received two or more chemotherapy regimens). It is noted that only three patients received trastuzumab as treatment for metastases. The average number of inpatient days (to death or to a maximum follow-up of 5 years) was 24, with an interquartile range of 5–34 days.

The following sections describe the cost results, as divided in the Materials and Methods section, for 5-year costs by recurrent event; annual costs for patients remaining alive in the same state; terminal-phase costs; and costs by time period. The results are presented using high–low–mean plots, where the line represents the 95% confidence interval, and the diamond represents the mean value.

### Aggregate 5-year costs

**Figure 2:**
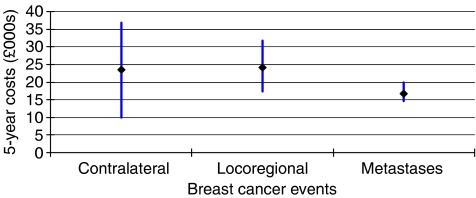
Five-year costs (with 95% confidence intervals) from diagnosis by first recurrent event.

Aggregate costs over a 5-year follow-up period for patients experiencing contralateral primary tumours, locoregional recurrence, and metastatic recurrence as their first recurrent breast cancer event are shown in Figure 2. It can be seen that patients whose first relapse is a metastatic recurrence have the lowest mean 5-year cost. There is relatively little uncertainty around the mean estimate of £16 640, though the confidence interval overlaps with the intervals for the other event types. The distribution of metastatic recurrence costs is positively skewed due to a small number of patients with very high costs. The main cause for the high costs in a few patients is extended inpatient stays; 14 of the 199 patients spent between 11 and 58 days in hospital in the year following their first recurrent event.

The estimated mean costs following a contralateral or locoregional event are similarly around £24 000, although the 5-year cost of contralateral tumours is subject to larger uncertainty due to the small number of contralateral cases observed (*n*=8). The upper 95% confidence intervals were estimated as £36 804 and £31 659 for contralateral and locoregional events, respectively.

### Annual costs for patients remaining alive without progression

**Figure 3:**
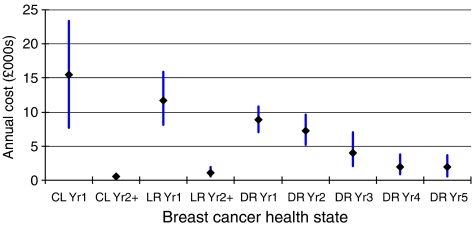
Mean annual costs (with 95% confidence intervals) for patients remaining alive in the same health state for a year.

Estimated annual costs for patients who experienced contralateral (CL), locoregional (LR), or distant recurrence (metastases) (DR), and remained in the same state for a year are shown in Figure 3. The results show that initial costs of treatment for contralateral primary tumours (mean £15 470, 95% confidence intervals £7 678–£23 355) are likely to be higher than the initial costs associated with locoregional recurrence (£11 701, £8070–£15 950). The confidence intervals overlap, with the large range for contralateral recurrences including the whole of the range for locoregional recurrence. In subsequent years, both contralateral and locoregional patients experiencing no further breast cancer event incur very few costs.

The data describing the costs of treating patients remaining alive for full years following distant recurrence display an obvious downwards trend. From a mean cost of £8825 (95% confidence interval £7076–£10 832) in the first year, costs decline at a steady rate in subsequent years to plateau at around £2000 per year in years 4 and 5 following diagnosis.

### Terminal-phase costs

**Figure 4:**
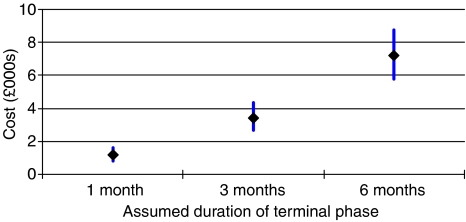
Mean terminal-phase costs (with 95% confidence intervals) for final 1, 3, and 6 months of life with metastases.

The separately estimated costs of providing palliative care to patients in the final months of life with metastases are presented in Figure 4. A terminal-phase duration of 3 months has a mean cost estimate of £3400, with an upper confidence interval of around £4300. If a longer terminal phase of 6 months is specified, the mean cost estimate rises to £7200, with an upper interval of almost £9000. For a 1-month phase, the mean estimate is more than £1000 with an upper interval of around £1600.

### Cost estimates by time period

Table 5 presents the first-year costs following diagnosis for the different recurrent events according to the time period in which the event occurred. The analyses are presented for the first year post-recurrence as these years had the largest samples. Analysis A includes all first-year post-recurrence patients, whereas analysis B includes only patients remaining alive and experiencing no further breast cancer event within the first year post-recurrence. The results are similar for both analyses with no evidence that the mean cost estimates differ across the time periods.

## DISCUSSION

This paper has analysed a unique dataset to estimate annual health care resource costs following a breast cancer recurrence. The analysis of the mean aggregate costs incurred to either death or a follow-up period of 5 years showed that patients experiencing a contralateral primary tumour or a locoregional recurrence incurred more costs than patients experiencing a distant recurrence without a prior contralateral or locoregional event. This potentially counterintuitive finding is explained by two factors. First, only around 10% of metastatic recurrence patients in this dataset lived for 5 years and hence most only incur costs over a small proportion of the 5-year time frame, while around 40% of locoregional patients lived till 5 years. Second, contralateral and locoregional events are associated with the high costs of further surgery and/or radical radiotherapy, combined with the fact that despite this radical approach, a high proportion of patients go on to experience distant recurrence within 5 years and hence incur the additional costs of treating metastatic disease.

The aggregate costs of recurrence are estimated to be high, in the region of £10 000–£40 000 for contralateral tumours and locoregional recurrence, and £15 000–£20 000 for distant recurrence. The analysis by time period found no evidence of significant variations in these costs across time periods, despite the fact that newer more expensive drugs have been licensed during the period of this study. One limitation though is that trastuzumab only became available towards the end of the years under consideration, as evidenced by the fact that only three of the patients received it. When evaluating the cost-effectiveness of newer, more effective but more expensive interventions for early breast cancer, the prevention of recurrence and the associated costs should at least partly offset the additional upfront costs of implementing new interventions.

A number of other studies have reported costs associated with different aspects of breast cancer care, ranging from estimates of specific items of resource use, such as that of Robertson *et al* (1995), who reported surveillance costs following a locoregional or a metastatic relapse to estimates of total costs following diagnosis of breast cancer at specific stages of the disease, primarily advanced breast cancer. Richards *et al* (1993) presented retrospective data on the aggregate cost of treatment for 50 patients with advanced breast cancer. The costs were presented in eight categories, and a total cost from diagnosis to death was calculated to be £7620 (1991 costs, £9120 when uprated to 2006 costs). Wolstenholme *et al* (1998) presented the mean 4-yearly costs of breast cancer treatment by stage. Taking stage IV as an example, a mean of zero was presented for inpatient stay investigations, and £72 (1991 costs) for inpatient stay complications, although a sample size of six restricts the use of such estimates.

More recent studies include that of Remak and Brazil (2004), who estimated the costs of managing stage IV breast cancer to be around £12 500 based on information about usual treatment practice that was collected from a survey of cancer physicians. Wolowacz *et al* (2005) estimated similar total costs following locoregional recurrence and metastases of around £13 500. The latter study was informed by a dataset of patients from Edinburgh. The Wolowacz's study included 188 node-positive patients who were given adjuvant chemotherapy and relapsed between 1992 and 2001, so there is partial overlap with the 199 relapses included in this study who were women aged 50 years or older and who had been treated using (at least) surgery and tamoxifen.

The cost estimates presented by the three most relevant previous costing studies undertaken in the United Kingdom are comparable with the results reported in the current study (Richards *et al*, 1993; Remak and Brazil, 2004; Wolowacz *et al*, 2005). With respect to the estimation of the aggregate costs of breast cancer recurrence, this study provides additional evidence of the magnitude of such costs.

The comparison with the studies reported by Richards *et al* and Remak and Brazil also supports the generalisability of the data reported in this study to other areas of the United Kingdom. Generalisability is important since one of the objectives of the current study was to estimate time- and state-specific costs of recurrence to populate cost-effectiveness models.

Cost-effectiveness models generally describe disease pathways between relevant health states to which unit costs are attached, encompassing the costs incurred by patients in each health state over a defined time period, typically 1 month or 1 year. The costs presented in this study, of remaining alive in the same state for 1 year, provide health economists and decision makers with an appropriate resource to populate cost-effectiveness models and to inform resource allocation decisions. Separate cost estimates for the first year following a contralateral primary tumour or a locoregional recurrence (in the absence of death or further recurrence) are presented. Costs in subsequent years after a contralateral or locoregional recurrence are shown to be small and relatively stable, therefore, a constant cost estimate can be applied to such health states. Costs associated with distant recurrence are shown to vary considerably between years, and so it may be more appropriate to apply separate annual costs out to 5-year post-metastatic recurrence.

The current study has two main limitations that should be considered when interpreting the presented results. First, the retrospective data describing the use of routine resource items, such as surveillance tests and outpatient visits were not linked to the timing of the breast cancer events experienced by patients. The data described total resource use in each full year following a patient's first recurrent event, which meant that a substantial amount of patient data had to be excluded from the annual resource costs as it was not clear whether some resource use was incurred before or after a further recurrence. This reduced the sample size, and, hence, the accuracy of the derived annual costs estimates, though additional analyses (not presented) undertaken around the specific resource items (hormonal therapy, chemotherapy, radiotherapy, and surgery), for which resource usage was linked to specific dates indicate that the estimated costs did not differ greatly between the two forms of analysis.

Second, the data are derived from a single UK location – a teaching hospital in Edinburgh. Issues around the generalisability of the presented cost estimates were addressed via the comparison with other UK costing studies. However, the cost estimates were limited to secondary health care resource use; costs of primary care and hospice care were not available for this cohort of patients. A broader dataset that incorporates primary care and hospice costs would be preferable though secondary care is likely to comprise the bulk of health care costs associated with the treatment of recurrent breast cancer.

Despite these limitations, the cost estimates presented in this paper provide additional evidence regarding the cost impact of recurrent breast cancer events. Moreover, they provide an important source to inform cost-effectiveness models of breast cancer interventions undertaken from a UK perspective. Previous UK costing studies were cost of illness studies, which may have a role in informing resource allocation to disease areas, where prevention or treatment have the greatest potential for reducing future health care expenditure. However, cost-effectiveness analysis plays a far greater role in allocating health care resources because it combines the cost impacts of alternative interventions with their health effects to enable informed and transparent trade-offs between interventions.

## Figures and Tables

**Table 1:** Surgery, radiotherapy, chemotherapy, and hormonal therapy unit costs

	**HRG**	**HRG description**	**Cost**
*Surgery*			
Excision of brain metastases	A03	Intracranial procedures except trauma – category 3	£5192
Excision of neck node	Q10	Procedures on the lymphatic system without cc	£1513
Lung resection	D06	Minor thoracic procedures	£573
Wide local excision	J06	Minor breast surgery with cc	£1261
Mastectomy	J02^a^	Major breast surgery including plastic procedures >49 or with cc	£2073
Mastectomy+axillary clearance	J02^a^	Major breast surgery including plastic procedures >49 or with cc	£2073
Oopherectomy	M07	Upper genital tract major procedures	£2398
Excision bone metastases	H22	Minor procedures to the musculoskeletal system	£800
Excision bone metastases with fixation	H22	Minor procedures to the musculoskeletal system	£800
Axillary node sample	Q10	Procedures on the lymphatic system without cc	£1513
Axillary node clearance	Q10	Procedures on the lymphatic system without cc	£1513
			
*‘Complex with imaging’ for radical radiotherapy to breast*			
	w13	Complex teletherapy with imaging, <4 fractions	£576
	w14	Complex teletherapy with imaging, >3 <13 fractions	£1119
	w15	Complex teletherapy with imaging, >12 <24 fractions	£1858
	w16	Complex teletherapy with imaging, >23 fractions	£2246
			
*‘Simple with simulator’ are suitable for palliative radiotherapy to metastases*			
	w06	Simple teletherapy with simulator, <4 fractions	£380
	w07	Simple teletherapy with simulator, >3 <13 fractions	£705
	w08	Simple teletherapy with simulator, >12 fractions	£1220
			
*Chemotherapy*			
	X01^a^	Breast cancer chemotherapy, CMFs	£220
	X02^a^	Breast cancer chemotherapy, anthracycline	£679
	X03^a^	Breast cancer chemotherapy, vinorelbine	£994
	X04^a^	Breast cancer chemotherapy, taxane	£3003
	X05^a^	Breast cancer chemotherapy, trastuzumab	£3764
	X06^a^	Breast cancer chemotherapy, other	£841
			
*Hormonal therapy*			
Megace	—	160 mg (BNF, 2005)	£0.98
Tamoxifen	—	20 mg (BNF, 2005)	£0.07
Anastrozole	—	1 mg (BNF, 2005)	£2.45
Letrozole	—	2.5 mg (BNF, 2005)	£2.97
Exemestane	—	25 mg (BNF, 2005)	£2.96
Goserelin^b^	—	3.6 mg (28 days) (BNF, 2005)	£84.14

cc=complications, comorbidities.

a NHS reference costs 2003 (uprated), all other HRG costs from NHS reference costs 2004.

b Breast cancer and prostate cancer by s.c. injection into anterior abdominal wall, 3.6 mg every 28 days.

**Table 2:** Routine tests, health service contacts, and treatments unit costs

**Resource item**	**HRG code**	**HRG description or other data source**	**Average unit cost**
Haematology	DAP823	—	£2.82
Chemical pathology	DAP822	—	£1.80
Plain films	J35op^a^	Minor radiology, includes plain film X-rays	£83
Bone scan	J25op^a^	Intermediate radiology	£155
Liver ultrasound	J33op^a^	Ultrasound scan	£123
CT	J23op^a^	MRI	£277
MRI	J24op^a^	CT	£177
FNA	J31op^a^	Fine needle aspiration with or without cytology	£119
Biopsy	J28op^a^	Excision biopsy	£123
Reconstruction	J01	Complex breast reconstruction using flaps	£4101
PIC/Hickman line	Q07^a^	Miscellaneous intermediate or minor vascular procedures (day case)	£616
Pleural effusion	D24	Pleural effusion without cc	£2,059
Biliary stenting	—	Personal communication: David Cameron, Edinburgh	£225
Blood transfusion	—	Varney and Guest, 2003	£652
Outpatient visit	370	Medical oncology	£125
Outpatient visit	800	Clinical oncology	£93
MDM+CBC^a^	—	Assumed to 3 × normal outpatient cost	£327
CBC pre-MDM	—	Assumed to 2 × normal outpatient cost	£218
IP nights neutropenic sepsis	—	Netten and Curtis, 2002 (uprated)	£365
Other IP nights	—	Netten and Curtis, 2002	£365
Bisphosphonates	—	Cost per month (BNF, 2005)	£27

CBC=combined breast clinic (three specialists); cc=complications, comorbidities; MDM=multidisciplinary meeting.

a NHS reference costs 2003 (uprated), all other HRG costs from NHS reference costs 2004.

**Table 3:** Patient characteristics

	**Total patients**	**Relapsed patients**
Number	816	199
*Age (years)*		
Median	60	59
Range	40–87	40–82
		
*ER*		
Rich	72.9%	66.3%
Poor	15.7%	24.6%
Not known	11.4%	9.0%
		
*Grade*		
1	15.3%	3.5%
2	34.1%	34.7%
3	21.0%	34.7%
Not known	29.6%	27.1%
		
*Number of positive nodes*		
0	69.6%	48.7%
1–3	22.3%	30.6%
4–9	4.2%	9.6%
10+	3.2%	11.1%
No axillary surgery	0.7%	0.0%
		
*Surgery*		
Wide local excision	80.8%	66.3%
Mastectomy	19.2%	33.7%
Adjuvant XRT	85.3%	83.4%
Adjuvant chemotherapy	14.1%	27.6%
Adjuvant tamoxifen	100%	100%

**Table 4:** Summary data for selected resources for patients with metastatic relapse

	**Number (%)**
Patients with distant recurrence	126 (100)
One hormonal therapy	55 (43.7)
Two or more hormonal therapies	18 (14.3)
One chemotherapy	32 (25.4)
Two or more chemotherapies	29 (23)
Herceptin	3 (2.4)
Radiotherapy	54 (42.9)
Bisphosphonates	36 (28.6)
Inpatient days (mean, IQR)	24 (5–34)

**Table 5:** Cost estimates across sequential time periods

	**Mean**	**Standard deviation**	**Sample**	**All periods (*F*-statistic)**	**Periods 2 and 3 (*t*-test)**
*Analysis A: first year costs (regardless of death or subsequent events)*					
*Contralateral tumour*					
Period 1 (1991–1995)	£12 238	£15 637	3	0.3749 (*P*=0.700)	—
Period 2 (1995–1999)	£18 906	£15 162	4		0.3725 (*P*=0.725)
Period 3 (1999–2004)	£23 528	£17 744	3		
					
*Locoregional recurrence*					
Period 1 (1991–1995)	£7376	£7675	8	1.5445 (*P*=0.231)	—
Period 2 (1995–1999)	£16 285	£15 798	20		1.005 (*P*=0.326)
Period 3 (1999–2004)	£8063	£7356	4		
					
*Metastases*					
Period 1 (1991–1995)	£9328	£6192	24	0.4175 (*P*=0.660)	—
Period 2 (1995–1999)	£9125	£7814	69		0.8590 (*P*=0.392)
Period 3 (1999–2004)	£10 398	£8738	56		
					
*Analysis B: first year costs (for patients remaining alive with no further breast cancer events)*					
*Contralateral tumour*					
Period 1 (1991–1995)	£12 238	£15 637	3	0.605 (*P*=0.567)	—
Period 2 (1995–1999)	£23 509	£14 705	3		1.1957 (*P*=0.271)
Period 3 (1999–2004)	£13 749	£10 003	6		
					
*Locoregional recurrence*					
Period 1 (1991–1995)	£6362	£3407	7	1.8744 (*P*=0.172)	—
Period 2 (1995–1999)	£11 422	£3414	13		1.2766 (*P*=0.214)
Period 3 (1999–2004)	£16 195	£13 026	12		
					
*Metastases*					
Period 1 (1991–1995)	£7077	£8431	5	0.1771 (*P*=0.839)	—
Period 2 (1995–1999)	£9059	£8334	35		0.2474 (*P*=0.805)
Period 3 (1999–2004)	£9409	£1602	36		
					
